# A Short History of the Development of Hospital Pharmacy in Belgium

**DOI:** 10.3390/pharmacy4030025

**Published:** 2016-09-01

**Authors:** Jean-Daniel Hecq

**Affiliations:** Hospital Pharmacy, CHU UCL Namur, Yvoir 5530, Belgium; jean-daniel.hecq@uclouvain.be; Tel.: +32-81-42-3300

**Keywords:** hospital pharmacy, clinical pharmacy, continuing professional development, continuing education health professions

## Abstract

The Belgian Association of Hospital Pharmacists (BAHP) is a professional and scientific association representing all pharmacists who work in hospital institutions, whether private or public, university, general or psychiatric. This association was created in 1953. The aim of this short paper is to tell the history of its continuous development in a few words. The main development is reviewed from 1950 to now including: regulation, professional association roles, agreement and continuing education, development of clinical pharmacy, and updating of university training program. A new decree for the hospital pharmacist is in the course of being finalized, including new technologies: automated dispensing, automated compounding, centralization of sterile compounding, e-learning, traceability of medical devices and clinical pharmacy.

## 1. Introduction

On 10 May 2013, the Belgian Association of Hospital Pharmacists (BAHP) celebrated 60 years of existence and became a Royal Society.

Created in 1953, this professional and scientific association represents all pharmacists who work in hospital institutions, whether private or public, university, general or psychiatric.

The aim of this short paper is to tell the history of this development in a few words.

## 2. A Continuous Development

### 2.1. Royal Decrees and Hospital Pharmacist Associations

Different royal decrees (RD) regulate the professional life of a Belgian hospital pharmacist. The first appeared in 1978 in the *Moniteur* [1], the official journal that publishes all laws and RDs, requiring the presence of a pharmacist in all hospitals of the Kingdom (RD of 19 October 1978) [2]. At the same time, the Belgian universities introduced a program of specialization in hospital pharmacy. From that moment, the profession began to differentiate from that of pharmacists working in community pharmacies. In 1986, the possession of this additional qualification for the right to practice in a hospital environment became compulsory. In 1989 two regional associations were created: one for Dutch speakers (the Flemish Association of Hospital Pharmacists—VZA) [3] and the other for French speakers (the French-speaking Association of Belgian Hospital Pharmacists—AFPHB) [4], following the federal trend within the Kingdom. These associations are represented with the BAHP [5].

The RD of 4 March 1991 fixes the standards which a hospital pharmacist must satisfy to be approved, which went on to significantly regulate the general and specific tasks of the pharmacist within his/her hospital institution, creating on the same occasion the term “Fonction hospitalière” in order to classify hospital pharmacy within it [6]. The pharmacist became a dispenser of drugs adapted to the needs of the patient [7].

Belgian hospital pharmacists are responsible for pharmaceutical specialties, extemporaneous preparations, antiseptics and disinfectants, registered dietary products, medical and surgical equipment, implants and prostheses, radio isotopes, medical gases and products that are under investigation by clinical trials.

The tasks are identical to pharmacists around the world: the individualized distribution of medicines, the preparation of non sterile and sterile medicines, the supply, stocking and appropriate storage of medicines, and the analysis and quality control of raw materials and medicines.

The specific tasks are very numerous: the organization of an effective, safe and economic system of distribution in the various hospital units; integration into multidisciplinary teams in order to optimize therapeutic efficacy and safety; the collection, processing and distribution, in a structured manner, of all the necessary pharmacological, toxicological and pharmaco-technical information concerning the medicines used in the hospital; active collaboration with the nursing staff regarding the use of medicines; the organization and promotion, in collaboration with the medical team, of pharmacovigilance activities; partial responsibility for the sanitary arrangements for hospitalized patients and discharged patients, in collaboration with the medical and nursing staff; making available for the hospital departments antiseptic and disinfectant solutions of an appropriate quality for the therapeutic necessities; guaranteeing at a qualitative level the daily activities of the sterilization unit; supervision of the galenical preparation of injectable solutions of radio-pharmaceuticals; drawing up, in collaboration with the doctor in charge of the hospital, an annual report, of the hospital as a whole and by department, of the consumption and cost of the medical treatment, and a report on the relation between the consumption of medicines and the pathologies treated in the hospital; contribution to clinical trials whenever they are performed in the hospital; contribution to the rapid and suitable treatment of cases of poisoning [8].

### 2.2. Agreement and Continuing Education

The particular title of hospital pharmacist was also created in 2003.

The RD of 11 June 2003 [9] describes the minimum program (900 h) of theoretical training and work experience. Theoretical training is divided into organization and hospital management, prevention and treatment of nosocomial infections, disease and drug therapy, hospital technology and radiopharmaceuticals.

For the extension of such recognition, the pharmacist must prove that he has obtained accreditation of 120 points during the validity period of its approval or over five years.

From the beginning of its creation, the Belgian association has organized training courses. These courses are then modeled on the matters described in RD of 2003.

Points can also be obtained by attendance at national and international conferences, presentation of oral communications, and publications of posters. That is why the Belgian delegation is often important to the congress of the European Association of Hospital Pharmacy and the European Society of Clinical pharmacy, for example [10,11].

## 3. The Start of Clinical Pharmacy

The development of clinical pharmacy began with isolated initiatives, mainly in university hospitals [12].

The first studies were published on geriatric inpatients [13].

In March 2007, Rudy Demotte, the Minister of Social Affairs and Public Health at the time, launched a call for clinical pharmacy projects from general and university hospitals in Belgium. This call for proposals was in response to ongoing efforts by the Belgian Association of Hospital Pharmacists to increase funding for clinical pharmacy in the country [14,15].

Proposals needed to specify the exact nature of a temporarily funded position, and on which ward or wards work would take place. Altogether, 80 proposals were received by the National Network of the Pharmaceutical and Therapeutic Committee (NN-PTC). Twenty-seven were selected for funding, which included 13 full-time and 15 half-time staff posts in clinical pharmacy in the areas of pain treatment, nutrition and seamless medication care in oncology, cardiology, intensive care units and geriatrics. Positions were filled, and the first evaluations of the projects by the hospitals were sent to the ministry by the end of the year. Two further reports were required before the end of the projects on 31 December 2008. The projects and accompanying reports should aid in demonstrating the positive impact of clinical pharmacists on patients and medical staff in a variety of areas, and hopefully serve as successful pilot projects for ongoing funding for more hospitals in Belgium. In January 2009, these 27 projects of clinical pharmacy were permanently established [16]. At the beginning of 2010, a new call for candidates was launched by the Minister of Public Health at the time, Laurette Onkelinx. More than 80 hospitals applied. Once again, 27 projects were selected and officially launched during an inaugural session in Brussels on 4 June 2010. The follow-up procedure by the National Network of the Pharmaceutical and Therapeutic Committee was the same as for the initial 27 projects. In 2010, 54 hospitals (26% of the number of hospitals in Belgium) had a clinical pharmacy project. Increasingly, clinical pharmacy forms part of the Belgian hospital landscape. In June 2014, the NN-PTC concluded that the experiences of the projects in place were conclusive. They decided, on the 1 July 2014, to inject a budget for 0.25 full-time equivalent (FTE) of clinical pharmacists per 200 beds in general and academic/university hospitals. A new plan is now in place to increase this budget once again in 2010 [17].

## 4. Updating of the University Training Program

The establishment of a specialization in hospital pharmacy in Belgium began in the late 1970s and consisted of three certificates: galenic formulation, pharmacology and hospital management. Since then, in other European countries, the duration of specialization studies has increased.

Furthermore, the content of the subjects taught and their diversity increased. Finally, the hospital pharmacist was involved in more and more areas and continuing education was not enough. An adaptation of study specialization was necessary.

In September 2010, a new training program for hospital pharmacists was established by all of the universities in Belgium. Sixty-five student posts were opened, 39 in the Flemish-speaking community and 26 in the French-speaking community [18,19]. The training comprises one academic part and four practical modules. The theoretical training contains organization and hospital management, nosocomial infection control, disease and drug therapy, hospital technology and radiopharmaceuticals.

The courses are taught by academic professors, lecturers, and ground health professionals.

The first practical module, entitled “Drugs supply chain”, includes the organization and management of a hospital pharmacy, the supply chain and the following topics: control processes and quality management, and information management on both drug and clinical trials.

Module 2, entitled “Hospital hygiene and central sterilization”, includes management of antibiotic therapy, organization and management of medical devices, supply chain and similar products (including activities relating to the remit of the Medical Devices Committee).

Module 3, entitled “Clinical pharmacy”, covers clinical pharmacy and pharmaceutical care in at least one ward (internal medicine, geriatrics, cardiology, pneumology, psychiatry, etc.) but also includes activities relating to the remit of the Pharmaceutical and Therapeutic Committee.

Module 4, entitled “Production”, includes the production and/or reconstitution of chemotherapy, radiopharmaceutical drugs and other preparations, including pharmaceutical and sterile preparations. These three parts must cover, in addition to the production itself, all control and quality assurance aspects.

The duration of each module is six months and the four modules are taken in sequence. The academic module is spread out over two years, at a rate of one day/week at the universities. This two-year program replaces the previous one-year program and is described in a written work which can be published. Currently, there is no English language version.

In September 2013, the program was extended to three years and the number of candidates was adjusted to 50. During the third year, the candidate may make the choice of one or two of the four modules described previously. The candidates are also strongly invited to do periods in other hospitals inside and outside Belgium. This third year will be ended on September 2016. Is it too early to make a conclusion at this time but the periods in another hospital met a big success with a preference for France and Quebec.

The candidates must also publish the results of their work as posters or publications. *Pharmakon*, the official journal of Belgian hospital pharmacy, has existed since 1969. For over 40 years, the Belgian hospital pharmacists were kept abreast of developments in their discipline by Dutch, French and sometimes English publications.

However, for many reasons (a lack of editorial board members, a lack of reviewers, too few articles and no access via Pubmed), the paper merged with the *Journal de Pharmacie* *de Belgique* in 2013. The publications of candidates and hospital pharmacists are now easily accessible [20,21].

## 5. Conclusions

The RD of March 1991 is outdated and must be updated. A new decree was published in 2009 concerning community pharmacists [22]. A new decree for hospital pharmacists is in the course of being finalized, including new technologies: automated dispensing, automated compounding, centralization of sterile compounding, e-learning, traceability of medical devices and clinical pharmacy.
